# Expression Characteristics of Gustatory Receptor Genes in *Galeruca daurica* (Coleoptera: Chrysomelidae) and Adult Behavioral and Electrophysiological Responses to Host Metabolites

**DOI:** 10.3390/insects17040442

**Published:** 2026-04-21

**Authors:** Jing Gao, Jinwei Li, Haichao Wang, Jinghang Zhang, Xiaomin An, Yanyan Li, Jun Zhao, Baoping Pang, Ling Li

**Affiliations:** 1 College of Horticulture and Plant Protection, Inner Mongolia Agricultural University, Hohhot 010018, China; gaojingww0509@163.com (J.G.); lijinwei611@163.com (J.L.); wanghc@imau.edu.cn (H.W.); liyanyan@imau.edu.cn (Y.L.); zhaojun@imau.edu.cn (J.Z.); pangbp@imau.edu.cn (B.P.); 2 Inner Mongolia Autonomous Region Plant Protection and Quarantine Center, Hohhot 010010, China; zhangjinghang0508@163.com; 3 Bayannur Modern Agriculture and Animal Husbandry Development Center, Bayannur 015000, China; bsnjtgzx@163.com

**Keywords:** *Galeruca daurica*, gustatory receptors, expression profiles, host-derived metabolites, single sensillum recording

## Abstract

*Galeruca daurica* (Joannis) (Coleoptera: Chrysomelidae) is an oligophagous pest in which both adults and larvae prefer to feed on *Allium* forage grasses of the Liliaceae family. In this study, we identified gustatory receptor (GR) genes based on the transcriptome data of *G. daurica*; analyzed the expression profiles of these GR genes across different larval instars and various tissues of male and female adults using quantitative real-time PCR (qRT-PCR); detected the electrophysiological responses of the mouthparts of male and female *G. daurica* adults to flavonoids and carbohydrates using single sensillum recording (SSR); and recorded the changes in food consumption of *G. daurica* adults after feeding on six host plant-derived metabolites. A total of 26 GR genes were identified from the transcriptome data of adult and larval of *G. daurica*. Phylogenetic analysis was performed to screen candidate functional gustatory receptor genes, including four sugar receptors (*GdauGR7*, *GdauGR10*, *GdauGR14* and *GdauGR28*), seven bitter receptors (*GdauGR11*, *GdauGR16~17*, *GdauGR22*, *GdauGR25~26* and *GdauGR30*), and two CO_2_ receptors (*GdauGR15* and *GdauGR20*). Larval expression profiling of *GdauGRs* in *G. daurica* revealed that the relative expression levels of 17 genes exhibited dynamic changes during larval growth and development. *GdauGRs* were expressed to varying degrees in the antennae, mouthparts, brain, gut, and forelegs of adult *G. daurica*, with sex-specific differences. Notably, the expression levels of *GdauGR4*, *GdauGR9* and *GdauGR16* in the gut were extremely significantly higher than those in other tissues. In the SSR test, the six tested flavonoids and one carbohydrate were able to induce robust electrophysiological responses in the gustatory sensilla on the antennae and mouthparts of adult *G. daurica* at specific concentrations. In addition, the supplementation of several host-derived metabolites altered the food consumption of adult *G. daurica*. These findings lay a solid foundation for elucidating the molecular mechanisms underlying gustatory recognition and host adaptation in *G. daurica*.

## Simple Summary

*Galeruca daurica* (Coleoptera: Chrysomelidae) is one of the major insect pests that have broken out and caused severe damage in the Inner Mongolia grassland in recent years, and it feeds exclusively on the leaves of *Allium* plants. This study for the first time constructed the spatiotemporal expression profile of gustatory receptor genes in *G. daurica*, primarily screening several effective chemical signal substances recognized by the gustatory system of *G. daurica*. These findings lay a solid foundation for elucidating the molecular mechanisms underlying gustatory recognition and host adaptation in *G. daurica*.

## 1. Introduction

The taste sensory system of insects plays a crucial role in various behaviors, such as foraging, oviposition, and avoidance of toxic substances. Gustatory receptors (GRs) are among the most important proteins in the gustatory system, which can efficiently and sensitively recognize non-volatile chemical cues. Chemical signal substances enter the gustatory sensilla and bind to GRs on the dendritic membranes of gustatory receptor neurons (GRNs), thereby generating electrical signals. These signals are transmitted to the central nervous system (CNS) in the form of action potentials and ultimately modulate insect behaviors [1]. In recent years, with the development of molecular biology and sequencing technologies, significant progress has been made in the research on insect taste perception, mainly focused on the encoding mechanisms of gustatory receptors in response to feeding and oviposition stimulants and deterrents [2,3]. For instance, in *Helicoverpa armigera*, larvae and adults perceive sucrose via Gr10 and Gr6, respectively, whereas Gr180 serves as the bitter receptor responsible for detecting coumarin, a feeding deterrent, in its larvae [4,5]. In *Pieris rapae*, Gr28 and Gr19 mediate the specific perception of distinct glucosinolates in Brassicaceae, respectively [6,7].

Herbivorous insects locate and analyze the chemical substances produced by plants through their chemical sensory systems, thereby determining whether to feed or avoid [8]. These chemicals are mainly derived from plant primary and secondary metabolism. Primary metabolites include carbohydrates, proteins, amino acids, and other compounds [9]. These substances provide essential nutrients for the growth and development of insects, and also play a certain role in promoting insect feeding [10]. Plant secondary metabolites are derived from primary metabolites. They can be classified into phenolic compounds including nitrogen-containing alkaloids and flavonoids, among others [11]. Unlike primary metabolites, they cannot provide nutrients to phytophagous insects, but can affect their feeding, growth, development, survival rate and other indicators [12].

*Galeruca daurica* (Joannis), a pest species belonging to Coleoptera (Chrysomelidae), has become a major pest causing severe outbreaks in the grasslands of Inner Mongolia, China, in recent years. According to historical records, *G. daurica* is mainly distributed abroad in Mongolia, Russia (Siberia), the Democratic People’s Republic of Korea and the Republic of Korea. In China, it is distributed in most grasslands of Inner Mongolia, and has also been recorded in Xinjiang and Gansu, posing a potential outbreak threat to grasslands in northern China [13]. As an oligophagous insect, both adults and larvae prefer to feed on *Allium* grasses of the Liliaceae family, such as *A. mongolicum* and *A. ramosum*, posing a serious threat to the sustainable development of grassland animal husbandry in China. Currently, the control of *G. daurica* mainly relies on chemical control. However, pesticide residues not only pose a potential risk to the safety of grassland livestock but also tend to bring huge economic losses to herdsmen. Therefore, an in-depth dissection of the molecular mechanisms underlying the specific recognition and feeding of *Allium* plants by *G. daurica* is conducive to identifying key targets for green prevention and control.

Based on the transcriptome data of *G. daurica* previously obtained in the laboratory, this study identified the gustatory receptor genes of *G. daurica*. The spatiotemporal expression profiles of these genes were analyzed using quantitative real-time polymerase chain reaction (qRT-PCR). Single sensillum recording (SSR) was employed to detect the electrophysiological responses of gustatory sensilla on the antennae and mouthparts of adults to 10 metabolites from the host plant. Meanwhile, feeding assays were conducted to evaluate the effects of six host-derived metabolites on the food consumption of adults. This study aims to provide a theoretical basis for elucidating the molecular mechanisms underlying the interaction between gustatory receptors and host chemical signals.

## 2. Materials and Methods

### 2.1. Test Materials

Larvae of *G.* *daurica* were collected from the grasslands of Siziwang Banner, Ulanqab City, Inner Mongolia, China (41.9354° N, 111.5914° E), and reared on fresh *A. mongolicum*. During the rearing period, 30 larvae at each instar stage and 30 newly emerged male and female adults (3 days post-eclosion) were collected. All samples were immediately frozen in liquid nitrogen and stored in a −80 °C ultra-low-temperature freezer. The host plant metabolites used in this study are listed in Table S1.

### 2.2. Identification and Bioinformatics Analysis of Candidate Gustatory Receptor Genes

Using “GR” and “Gustatory receptor” as the key words, the annotation information of all-unigenes from the transcriptome data of adult and larval *G. daurica* previously assembled in the laboratory was searched to initially screen out candidate gustatory receptor genes. To confirm the reliability of the candidate genes, all candidate GR sequences were individually subjected to Blastx homologous sequence alignment against the NR database on the NCBI website (https://blast.ncbi.nlm.nih.gov/, accessed on 4 March 2023), with an e-value threshold set at 10^−5^. Subsequently, the Open Reading Frames (ORFs) of all candidate GR genes were predicted using the NCBI online tool ORF Finder (https://www.ncbi.nlm.nih.gov/orffinder/, accessed on 4 March 2023).

### 2.3. Phylogenetic Analysis of Gustatory Receptor Genes in G. daurica

A phylogenetic tree of GRs was constructed using the neighbor-joining (NJ) method in MEGA 11 software. The Jones–Taylor–Thornton (JTT) model was adopted for 1000 bootstrap replicates, and only bootstrap support values greater than 50% were displayed. The phylogenetic tree encompasses 18 insect species from 4 orders, with a total of 114 amino acid sequences of gustatory receptors. Specifically, there were 82 sequences from 11 Coleoptera species, including 26 sequences from *G. daurica* (GdauGR), 14 sequences from *Pyrrhalta aenescens* (PaenGR), 6 sequences from *Pyrrhalta maculicollis* (PmacGR), and others. For Hymenoptera, 7 sequences from 2 species were included, namely 6 sequences from *Apis mellifera* (AmelGR) and 1 sequence from *Camponotus floridanus* (CfloGR). Regarding Lepidoptera, 8 sequences from 2 species were involved, including 7 sequences from *Bombyx mori* (BmorGR) and 1 sequence from *Helicoverpa armigera* (HarmGR). For Diptera, 17 sequences from 3 species were included, which were 13 sequences from *Drosophila melanogaster* (DmelGR), 3 sequences from *Anopheles gambiae* (AgamGR), and 1 sequence from *Aedes aegypti* (AaegGR).

### 2.4. Expression Profile Analysis of Gustatory Receptor Genes in Different Instars of G. daurica Larvae

Total RNA was extracted from *G. daurica* larvae at different instars using the Eastep^®^ Super Total RNA Extraction Kit (Promega, Madison, WI, USA). The RNA concentration was determined with a microspectrophotometer NanoPhotometer^®^ P330 (Implen, Munich, Germany). RNA was reverse-transcribed into cDNA using the PrimeScript RT Reagent Kit with gDNA Eraser (Perfect Real Time) (Takara, Dalian, China), and cDNA was used as the template for quantitative real-time PCR (qPCR). Succinate dehydrogenase (SDHA) was used as the reference gene, and qPCR primers were designed using the online software Primer 3 Input (https://bioinfo.ut.ee/primer3-0.4.0/, accessed on 21 March 2023). All primers were synthesized by Sangon Biotech (Shanghai) Co., Ltd. (Shanghai, China), and the primer sequences are shown in Table S2. Due to the short sequence of GdauGR19, qPCR primers could not be designed, so only the other 25 GR genes were subjected to qPCR analysis.

The qPCR reaction program was set as follows: ① pre-denaturation at 95 °C for 10 min; ② denaturation at 95 °C for 15 s, annealing at 60 °C for 1 min, and extension at 72 °C for 30 s, with step ② repeated 40 times. The expression level of *GdauGR1* in 1st instar larvae was used as the control among different larval instars. Each sample was subjected to three biological replicates and three technical replicates. After the reaction, Ct values were obtained, data analysis was performed using the 2^−ΔΔCt^ method, and graphs were plotted with GraphPad Prism 10.0.

### 2.5. Expression Profile Analysis of Gustatory Receptor Genes in Different Tissues of Male and Female Adult G. daurica

The antennae, mouthparts, brains, intestines, and forelegs of male and female adult *G. daurica* were dissected using ophthalmic scissors and forceps. The qPCR reaction program was set as follows: ① pre-denaturation at 94 °C for 30 s; ② denaturation at 94 °C for 5 s, annealing at 60 °C for 30 s, with step ② repeated 40 times. The expression level of each gene in the antennae of female adults was used as the control. The other test contents were the same as those in Section 2.4.

### 2.6. Electrophysiological Effects of 10 Substances from A. mongolicum on Adult G. daurica via Single Sensillum Recording (SSR)

Based on the metabolites of carbohydrates and flavonoids in *A. mongolicum* detected previously in our laboratory (Tables S3 and S4), combined with relevant literature reports [14,15], 10 major metabolites were selected from *A. mongolicum*. These substances included 6 flavonoids (Prunin, PRU; Scutellarin, SCU; Narcissin, NAR; Rutin, RUT; Isoflavone, ISO; Isoquercetin, IQC) and 4 carbohydrates (Phenyl-β-D-glucopyranoside, PBG; Trehalose, TRE; D-Galactose, Gal; L-Rhamnose, Rha). SSR was conducted using a Syntech recording system (Hilversum, The Netherlands) to examine the electrophysiological responses of male and female adults. Each substance was set with three concentration gradients (specific concentrations are shown in Table S5), 50% ethanol was used as the solvent, and at least five adults were tested for each treatment group.

*G. daurica* adults at 3rd day post-eclosion were selected for the experiment. After being starved for 12 h, their bodies were excised, with only the head retained. One end of a silver wire electrode was gently inserted into the head incision, and the other end was sheathed with a glass capillary filled with the test solution. A micromanipulator was used to place the glass capillary over the tip of the gustatory sensilla and the circuit was activated by lightly stepping on the foot pedal, with a reaction time of 5 s. The response potentials were recorded using Autospike 32 software (Syntech, Hilversum, The Netherlands), and the interval between each operations was at least 2 min to avoid sensory adaptation.

### 2.7. Effects of 6 Substances from A. mongolicum on Food Consumption of G. daurica Adults

In the feeding experiments conducted on adult *G. daurica*, given that our laboratory had previously carried out behavioral assays on four compounds (ISO, IQC, Gal, and Rha), only the remaining six compounds were investigated in the present study. These six compounds were selected as dietary additives for the feeding assays conducted on adult beetles, including four flavonoids (PRU, SCU, NAR, RUT) and two carbohydrates (PBG and TRE). Two concentration gradients were set for each substance (specific concentrations are shown in Table S6), with distilled water used as the solvent. For each treatment, 30 adults individuals at 3rd day post-eclosion were selected and randomly assigned to five rearing containers, with six individuals per container. They were fed daily with *A.* *mongolicum* soaked in the corresponding test solution. Food consumption was recorded continuously for 10 days. A volatile control group was set up simultaneously each day to minimize experimental errors.

The calculation formulas were as follows:

Volatilization rate (%) = (Initial weight of *A. mongolicum* − Residual weight after 24 h)/Initial weight × 100%

Food consumption (mg) = Initial weight of *A. mongolicum* × Volatilization rate − Residual weight

Net difference in food consumption (mg) = Food consumption of the treatment group − Food consumption of the control group

## 3. Results

### 3.1. Identification and Bioinformatics Analysis of GdauGR Genes

A total of 30 putative gustatory receptor genes were identified from the transcriptome data of *G. daurica*, designated as *GdauGR1~30* (GenBank: PZ2558813–PZ2558842). After sequence alignment, nucleotide sequence overlaps were identified between *GdauGR2* and *GdauGR21*, *GdauGR5* and *GdauGR10*, *GdauGR9* and *GdauGR27*, and *GdauGR8* and *GdauGR12*. Thus, *GdauGR5*, *GdauGR8*, *GdauGR21* and *GdauGR27* were excluded from subsequent analyses.

BLASTX alignment results (Table 1) showed that all genes obtained high-confidence functional annotations, and their best matches were mainly derived from Coleoptera insects, including *Pyrrhalta aenescens*, *Diabrotica virgifera virgifera*, *Anoplophora glabripennis* and others. This indicated that these genes are evolutionarily highly conserved. The ORFs of the 26 *GdauGRs* genes were from 117 to 1242 bp in length, encoding 38 to 413 amino acids, with varying degrees of deletions at both ends of the sequences.

### 3.2. Phylogenetic Analysis of Gustatory Receptor Genes in G. daurica

**Figure 1:**
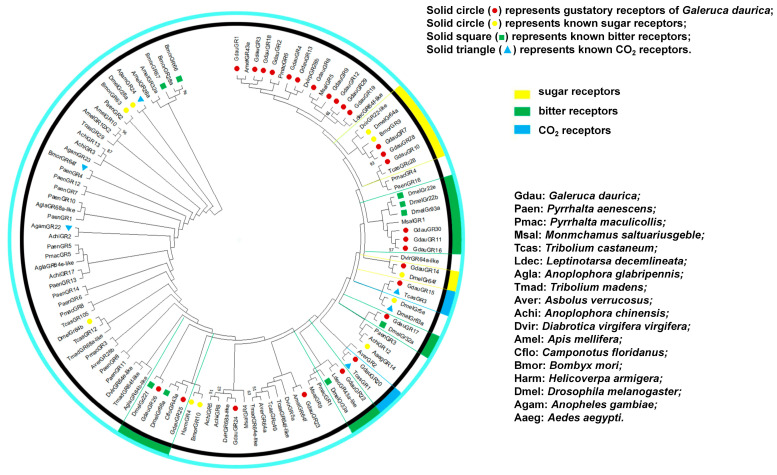
Phylogenetic tree based on amino acid sequences of GR from *G. daurica* and other insects.

Phylogenetic tree analysis results showed that *GdauGR12* and *GdauGR29*, *GdauGR7* and *GdauGR28*, as well as *GdauGR11* and *GdauGR16* were clustered into the same small branches, with bootstrap support values of 61%, 93%, and 57%, respectively, indicating that they share high sequence homology (Figure 1). Some *GdauGRs* were clustered into the same clades as several gustatory receptors with known functions. For example, *GdauGR7*, *GdauGR10*, and *GdauGR28* were clustered into the same clade with the sugar receptors *DmelGR64a* from *Drosophila melanogaster* and *BmorGR9* from *Bombyx mori*; and *GdauGR14* clustered into one clade with the sugar receptor *DmelGR64f*. *GdauGR11*, *GdauGR16*, and *GdauGR30* were clustered into one clade with the bitter receptors *DmelGR22b*, *DmelGR22e*, and *DmelGR93a* from *Drosophila*; *GdauGR17* was clustered together with the bitter receptor *DmelGR32a*; *GdauGR22* was clustered together with the bitter receptor *DmelGR33a*; and *GdauGR25* and *GdauGR26* were clustered into the same clade with the bitter receptors *DmelGR22f* and *DmelGR66a*. *GdauGR15* and *GdauGR20* were clustered into one clade with the CO_2_ receptors *TcasGR3* and *TcasGR1* from *Tribolium castaneum*, respectively. We speculate that these receptors may possess potential corresponding functions. Therefore, we classified these gustatory receptors of *G. daurica* functionally: sugar receptors (*GdauGR7*, *GdauGR10*, *GdauGR14*, *GdauGR28*); bitter receptors (*GdauGR11*, *GdauGR16*, *GdauGR17*, *GdauGR22*, *GdauGR25*, *GdauGR26*, *GdauGR30*); and CO_2_ receptors (*GdauGR15*, *GdauGR20*). Since the obtained gene sequences are incomplete, this functional classification only provides a reference for studying the actual functions of various gustatory receptors in *G. daurica*.

### 3.3. Expression Profile Analysis of GdauGRs at Different Larval Instars of G. daurica

**Figure 2:**
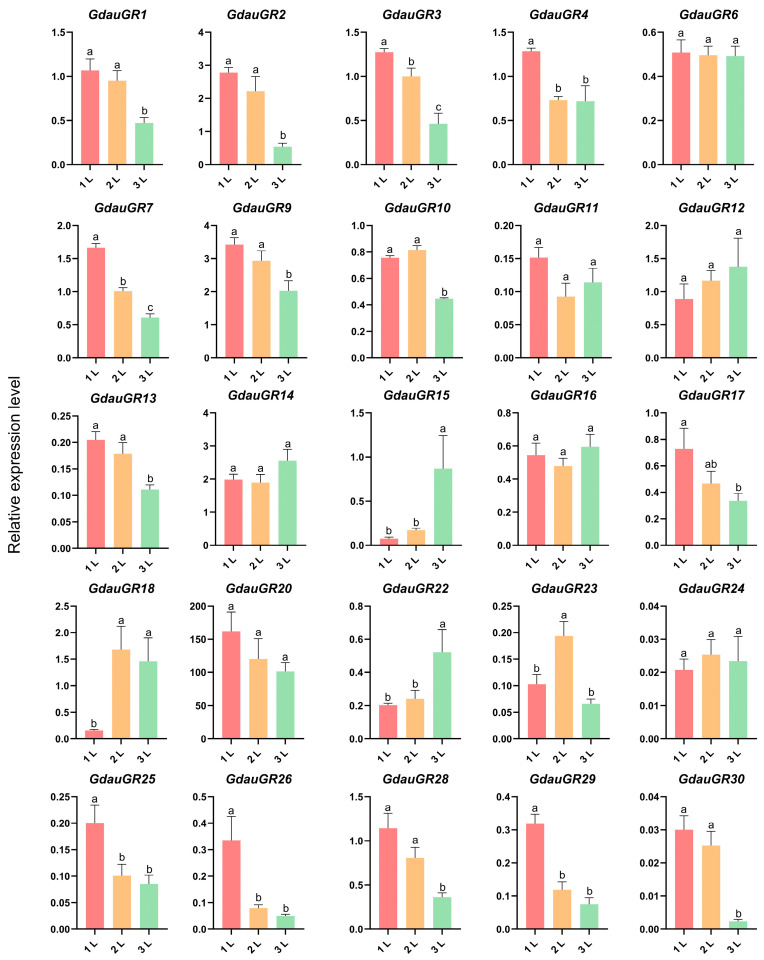
Expression profiles of *GdauGRs* in different instars of larvae. Note: 1 L: 1st instar larva; 2 L: 2nd instar larva; 3 L: 3rd instar larva. The expression level of GdauGR1 in 1st instar larvae was used as the reference. One-way analysis of variance (ANOVA) was adopted for statistical analysis with Duncan’s multiple range test; different letters indicate significant differences (*p* < 0.05). Error bars represent the standard error of three biological replicates.

The qRT-PCR analysis revealed (Figure 2) that the relative expression levels of 14 genes decreased with the progression of larval instars, including *GdauGR1~4*, *GdauGR7*, *GdauGR9~10*, *GdauGR13*, *GdauGR17*, *GdauGR25~26* and *GdauGR28~30*. Among them, the relative expression levels of *GdauGR1~2*, *GdauGR9~10*, *GdauGR13*, *GdauGR28* and *GdauGR30* were significantly higher in the first and second instar larvae than in the third instar larvae. The relative expression levels of *GdauGR3~4*, *GdauGR7*, *GdauGR25~26* and *GdauGR29* were significantly higher in the first instar larvae than in the second and third instar larvae. In contrast, the relative expression levels of the three genes *GdauGR15*, *GdauGR18* and *GdauGR22* increased with the progression of larval instars. Among them, the relative expression levels of *GdauGR15* and *GdauGR22* were significantly higher in third instar larvae than in first and second instar larvae. The relative expression level of *GdauGR18* was significantly higher in second and third instar larvae than in first instar larvae. In addition, the relative expression levels of seven genes showed no significant differences during the growth and development of larvae (*p* > 0.05), including *GdauGR6*, *GdauGR11~12*, *GdauGR14*, *GdauGR16*, *GdauGR20,* and *GdauGR24*. Notably, the relative expression level of *GdauGR20* was consistently high across all larval instars. The expression levels in first, second and third instar larvae were 162.07-fold, 120.26-fold and 101.26-fold that of the control group, respectively.

### 3.4. Expression Profile Analysis of GdauGR in Different Tissues of Male and Female Adult G. daurica

**Figure 3:**
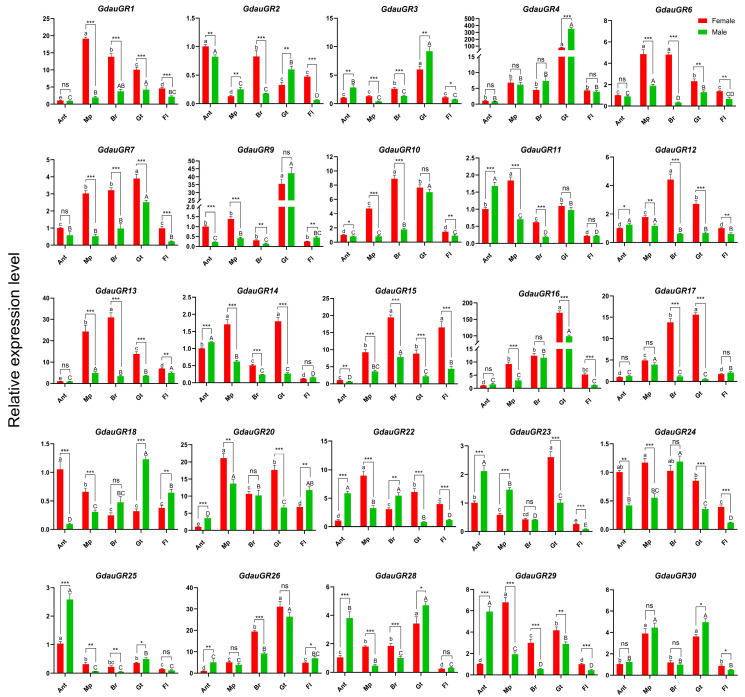
Expression profiles analysis of *GdauGRs* in different tissues of adult. Note: Ant: Antenna; Mp: Mouthpart; Br: Brain; Gt: Gut; Fl: Foreleg. The expression level in the antennae of female adults was used as the reference. Error bars represent the standard error of three biological replicates. Different lowercase and uppercase letters indicate significant differences among different tissues in female and male adults, respectively. One-way analysis of variance (ANOVA) with Duncan’s new multiple range test was used for statistical analysis, where different letters denote significant differences (*p* < 0.05). Asterisks above the bars indicate significant differences between female and male adults in the same tissue (***: *p* < 0.001; **: *p* < 0.01; *: *p* < 0.05; ns: no significant difference; *t*-test).

The qRT-PCR results of the target genes in various tissues of adult *G*. *daurica* demonstrated that all GR genes were expressed at varying levels across different adult tissues (Figure 3). For example, *GdauGR2*, *GdauGR11*, *GdauGR14*, *GdauGR18*, *GdauGR23*, *GdauGR25,* and *GdauGR29* showed relatively high expression levels in antennae; *GdauGR1*, *GdauGR6*, *GdauGR11*, *GdauGR14*, *GdauGR20*, *GdauGR22,* and *GdauGR29~30* exhibited relatively high expression levels in mouthparts; *GdauGR6*, *GdauGR10*, *GdauGR12~13*, *GdauGR15,* and *GdauGR24* had relatively high expression levels in brain; and *GdauGR3~4*, *GdauGR7*, *GdauGR9~10*, *GdauGR14*, *GdauGR16*, *GdauGR17~18*, *GdauGR23*, *GdauGR26*, *GdauGR28,* and *GdauGR30* showed relatively high expression levels in gut. All the tested genes presented low relative expression levels in the forelegs.

In addition, the relative expression levels of most genes exhibited sex-specific differences across different tissues of adult *G*. *daurica*. Among them, multiple genes exhibited significantly higher expression levels in multiple tissues of female adults than in male adults (*p* > 0.05), including *GdauGR1*, *GdauGR6~7*, *GdauGR10*, *GdauGR12~13*, *GdauGR15~16*, *GdauGR24* and *GdauGR29*.

Notably, *GdauGR4*, *GdauGR9* and *GdauGR16* exhibited significantly higher expression levels in the gut of both female and male *G*. *daurica* adults than in other tissues (*p* < 0.001). Among them, the expression level of *GdauGR4* was significantly higher in the gut of male adults than in that of female adults, and was 341.49-fold and 73.71-fold that of the control group, respectively. In contrast, the expression level of *GdauGR16* was significantly higher in the gut of female adults than in that of male adults, and was 165.25-fold and 95.71-fold that of the control group, respectively. Meanwhile, *GdauGR9* showed no significant difference in relative expression level in the gut of female and male adults (*p* > 0.05).

### 3.5. Electrophysiological Effects of 10 Substances from Allium mongolicum on Male and Female Adult G. daurica via Single Sensillum Recording (SSR)

**Figure 4:**
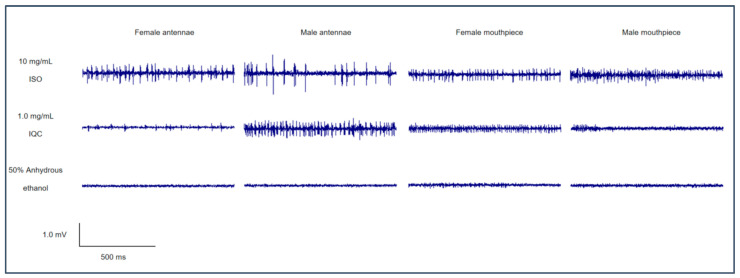
Representative traces of electrophysiological response of gustatory sensilla in male and female adult *G. daurica*.

**Figure 5:**
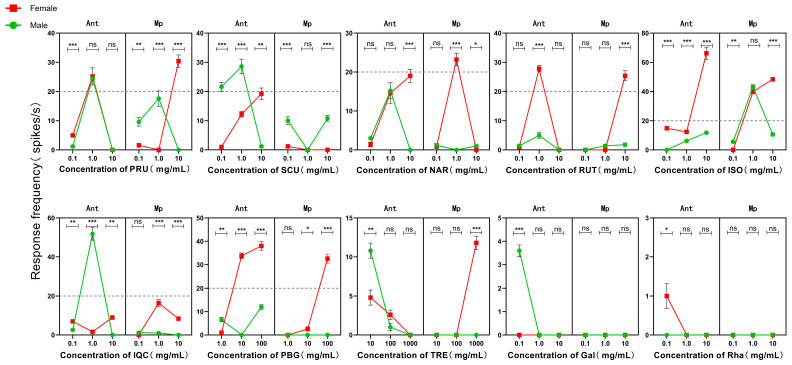
Electrophysiological response of Gustatory sensilla in male and female adult *G. daurica*. Note: Ant: Antenna; Mp: Mouthpart. Error bars represent the standard error of five biological replicates. Asterisks above the bars indicate significant differences between female and male adults at the same concentration (***: *p* < 0.001; **: *p* < 0.01; *: *p* < 0.05; ns: no significant difference; *t*-test).

SSR analysis results revealed that among the 10 tested compounds, 6 flavonoids and 1 carbohydrate could elicit relatively high potential responses from the gustatory sensilla on the antennae and mouthparts of adult *G. daurica* at specific concentrations (Table 2, Figure 4 and Figure 5). Responses with a firing frequency higher than 20 spikes/s were defined as strong responses. Among these, isoflavone was the most potent stimulant. At a concentration of 10 mg/mL, the electrophysiological response frequency of gustatory sensilla on the antennae of female adults reached the maximum at 66.20 spikes/s, which was significantly higher than those at other concentrations, and significant differences were observed between female and male adults. At this concentration, the response frequency of gustatory sensilla in the mouthparts of female adults was 48.40 spikes/s, also significantly higher than that at other concentrations and with a significant difference between males and females. Next, at a concentration of 1.0 mg/mL, the response frequencies of gustatory sensilla in the mouthparts of female and male adults were also relatively high, at 39.80 and 43.20 spikes/s, respectively, and no significant differences were found between the two sexes.

At a concentration of 1.0 mg/mL, prunin elicited a strong stimulatory response from the gustatory sensilla on the antennae, with response frequencies of 25.2 and 24.6 spikes/s in female and male adults respectively and no significant differences observed between the two sexes. In contrast, for the gustatory sensilla in the mouthparts, the response of female adults peaked at a concentration of 10 mg/mL and a frequency of 30.4 spikes/s, which was significantly higher than those at other concentrations. Furthermore, significant sexual differences were detected. When stimulating the gustatory sensilla on the antennae, scutellarin induced a maximum response frequency of 28.6 spikes/s in male adults at 1.0 mg/mL, with a significant difference between female and male adults; therefore, the gustatory sensilla in the mouthparts showed low or no response to scutellarin. At a concentration of 1.0 mg/mL, narcissin elicited a relatively high electrophysiological response from the gustatory sensilla in the mouthparts of female adults, with a frequency of 23.2 spikes/s. Rutin triggered a relatively strong response from the gustatory sensilla on the antennae of female adults at 1.0 mg/mL (27.8 spikes/s), and induced a relatively high electrophysiological response from the gustatory sensilla in the mouthparts of female adults at 10 mg/mL with a frequency of 25.4 spikes/s. When the gustatory sensilla were stimulated with isoquercetin, only the antennae of male adults exhibited a strong response to the 1.0 mg/mL concentration with a frequency of 51.8 spikes/s.

Phenyl-β-D-glucopyranoside could elicit a relatively high electrophysiological response from the gustatory sensilla on the antennae of female adults at a concentration of 10 mg/mL (33.8 spikes/s). At a concentration of 100 mg/mL, it induced relatively high electrophysiological responses from the gustatory sensilla on the antennae and in the mouthparts of female adults, with response frequencies of 38.00 spikes/s and 32.60 spikes/s, respectively. The gustatory sensilla on the antennae and in the mouthparts of *G. daurica* exhibited no or low electrophysiological responses to the other tested carbohydrate compounds, including trehalose, D-galactose, and L-rhamnose.

### 3.6. Effects of Six Substances from A. mongolicum on Food Consumption of G. daurica Adults

**Figure 6:**
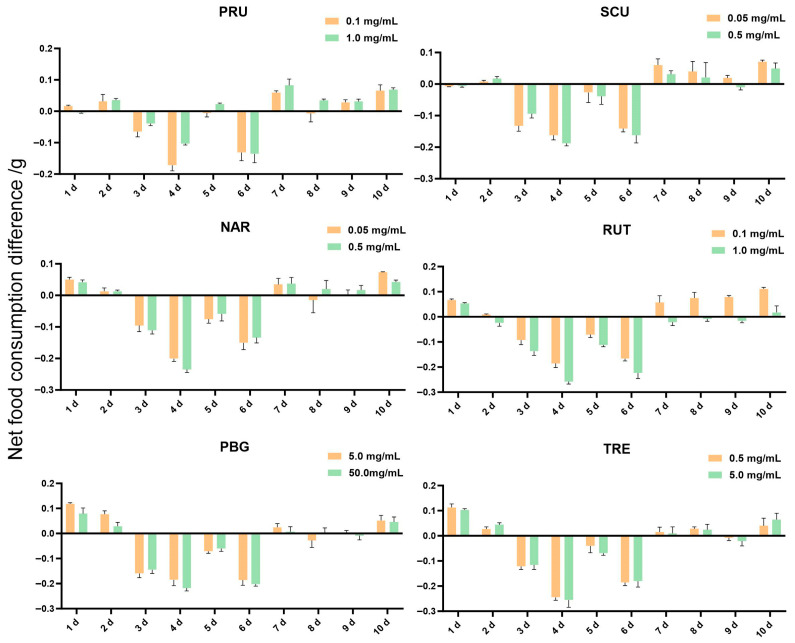
Influence of 6 chemical substances on food intake of female adults of *G. daurica*. Note: Data in the table are mean ± SD.

We added six host-derived metabolites, including four flavonoids (prunin, scutellarin, narcissin, rutin) and two carbohydrates (Phenyl-β-D-glucopyranoside, trehalose), to the diet of adult *G.* *daurica.* Food consumption was recorded over a 10-day period (Figure 6). The results showed that the net food consumption difference in all treatment groups exhibited phased fluctuations. On days 1~2, the net food consumption difference was positive in most treatment groups; from days 3 to 6, it became negative in all groups, indicating feeding inhibition; and from days 7 to 10, the inhibitory effect generally disappeared, and the net food consumption difference in most treatment groups returned to positive values.

## 4. Discussion

In recent years, with the rapid development of sequencing technologies and bioinformatics, an increasing number of insect gustatory receptors have been identified. For instance, 21 GR genes were identified in *Bombus impatiens* [16]; 26 GR genes were identified in *Spodoptera litura* [17]; 67 GR genes were identified in *Plutella xylostella* [18]; 76 GR genes were identified in *Bombyx mori* [19]; and 197 GR genes were identified in *Helicoverpa armigera* [20]. In this study, 26 GR genes were identified from the transcriptome data of adults and larvae of *G. daurica*. Phylogenetic analysis was performed to screen candidate functional gustatory receptor genes, including four sugar receptors (*GdauGR7*, *GdauGR10*, *GdauGR14* and *GdauGR28*), seven bitter receptors (*GdauGR11*, *GdauGR16~17*, *GdauGR22*, *GdauGR25~26* and *GdauGR30*), and two CO_2_ receptors (*GdauGR15* and *GdauGR20*). However, the incompleteness of most sequences may result in the loss of key conserved sites, which will reduce the reliability of phylogenetic analysis to a certain extent [21]. The number of gustatory receptor genes varies considerably among different insect species, which is mainly associated with multiple factors such as insect feeding habits, environmental conditions, and the complexity of host plants. Polyphagous insects usually require a larger number of gustatory receptor genes to recognize a wider range of chemical compounds [22]. The relatively small number of gustatory receptor genes in *G. daurica* may be primarily attributed to its oligophagous feeding habit. Meanwhile, transcriptome data only capture the genes expressed in specific tissues of a species under particular experimental conditions and environmental contexts. Therefore, subsequent studies can detect the whole genome of *G. daurica* to comprehensively obtain the complete data of the GR gene family, which will provide a solid data foundation for further in-depth research.

Gustatory receptors play a crucial role in the process of host plant selection and avoidance in insects. Investigating the expression patterns of insect gustatory receptors across different developmental stages and tissues can facilitate the inference of their functional roles [23]. Larval expression profiling of *GdauGRs* in *G. daurica* revealed that the relative expression levels of 17 genes exhibited dynamic changes during larval growth and development. Among them, the expression levels of 14 genes decreased as larval instars progressed, while those of 3 genes increased with advancing instar stages. This may be associated with the differences in nutritional requirements and metabolic intensity of larvae at different instars, and some genes may be involved in the recognition and utilization of specific nutrients. For instance, the majority of GR genes in *Bactrocera minax* showed relatively high expression levels in first and second instar larvae, promoting larval growth by sensing hesperidin and naringin [24]. The bitter taste receptor *BmGr63* of *Bombyx mori* is highly expressed in the mouthparts of fourth and fifth instar larvae, and can specifically recognize isoquercetin, a feeding stimulant in mulberry leaves. Loss of its function leads to a decrease in food consumption in *Bombyx mori* [25]. Notably, the relative expression level of *GdauGR20* remained high in all larval instars, suggesting that it plays an important role in the larval feeding of *G*. *daurica*.

It has been reported that in addition to being predominant expression in gustatory organs such as the labella, pharynx and legs of insects, GR genes are also expressed in the antennae, thorax, abdomen, enteroendocrine cells and reproductive organs [26,27,28]. In this study, *GdauGRs* were expressed to varying degrees in the antennae, mouthparts, brain, gut, and forelegs of adult *G.* *daurica*, with sex-specific differences, indicating that they are involved in diverse physiological and behavioral processes in this species. Notably, the expression levels of *GdauGR4*, *GdauGR9* and *GdauGR16* in the gut were extremely significantly higher than those in other tissues, indicating that gustatory genes are not only involved in insect feeding selection but may also directly participate in intestinal nutrient sensing, regulation of digestive metabolism, or interaction with gut microbiota in the intestine. This is consistent with the function of “intestinal taste receptors” reported in *B.* *mori* in recent years [29,30], and also provides novel insights for subsequent experiments.

In the SSR test, the six tested flavonoids and one carbohydrate were able to induce robust electrophysiological responses in the gustatory sensilla on the antennae and mouthparts of adult *G. daurica* at specific concentrations. This indicated that these substances are effective signaling molecules for gustatory perception in *G. daurica*, and that the corresponding sensory neurons are present in the gustatory sensilla on the antennae and mouthparts of its adults. The chemosensory functional specialization of insect mouthparts and antennae is closely related to their gene expression profiles. Sex- and tissue-specific gene expression directly determines the differential recognition of various chemical compounds between female and male adults [31]. Prunin can elicit strong electrophysiological responses in sensilla on the antennae of both male and female adults and on the mouthparts of female adults, which is consistent with the expression profile of *GdauGR14* in the antennae and mouthparts of adults. Therefore, *GdauGR14* may act as the receptor responsible for the recognition of prunin. Scutellarin and isoquercetin only elicited strong electrophysiological responses on male antennae, whereas *GdauGR25* and *GdauGR28* were significantly highly expressed in male antennae according to the expression profile. We speculate that these two genes may be the key receptors enabling males to respond to scutellarin and isoquercetin. Narcissin only triggered strong electrophysiological responses in sensilla on female mouthparts. Gustatory receptors specifically highly expressed in female mouthparts included *GdauGR1*, *6–7*, *10*, *13*, and *GdauGR15–16*, which may be involved in narcissin recognition. Rutin and phenyl-β-D-glucopyranoside induced strong electrophysiological responses in sensilla on female antennae and mouthparts. Combined with the preferential expression of *GdauGR9*, *18*, and *GdauGR24* in female antennae and mouthparts, we infer that these three genes may be the key receptors for females to recognize rutin and phenyl-β-D-glucopyranoside.

Supplementing several host-derived metabolites in the diet of *G. daurica* altered adult food consumption, showing slight feeding stimulation in early and late days, while high inhibition in the middle stage. The mild feeding stimulation during the early exposure stage could be attributed to exploratory behavior or low-dose hormesis, in which certain metabolites transiently activate gustatory pathways [6,32]. The significant feeding inhibition observed from day 3 onward may result from the progressive accumulation of toxic compounds, leading to dysfunction of the digestive or nervous system [5], accompanied by insufficient detoxification capacity despite the induced expression of enzymes such as P450s, glutathione S-transferases (GSTs), and esterases [33]. However, the subsequent recovery of feeding behavior may be explained by two mechanisms: (1) detoxification enzymes are eventually upregulated to metabolically effective levels; (2) adaptive changes occur in the gut microbiota to enhance detoxification ability. Studies have shown that plant secondary metabolites can induce the production of reactive oxygen species (ROS) in the midgut of Spodoptera frugiperda, activate the ROS/CncC-MafK signaling pathway, and further upregulate the expression and enzymatic activity of multiple detoxification genes including P450, GST, and UGT, ultimately conferring tolerance to various insecticides [34]. In the preliminary studies of our laboratory, the supplementation of host-derived metabolites in the diet of larvae altered the ribosomal and metabolic pathways of *G. daurica*, indicating that this pest may adapt to host plants by synthesizing corresponding proteins and accelerating metabolic processes [35].

This study for the first time constructed the spatiotemporal expression profile of gustatory receptor genes in *G. daurica* and screened several effective chemical signal substances recognized by the gustatory system of *G. daurica*. These findings lay a solid foundation for elucidating the molecular mechanisms underlying gustatory recognition and host adaptation in *G. daurica*. In future research, we will further investigate the functions of gustatory receptors in *G. daurica* through experiments such as immunofluorescence localization and RNA interference.

## Figures and Tables

**Table 1:** List of GRs in *G. daurica* transcriptome.

Gene Name	5′/3′ End Integrity	Length (bp)	Number of AA	Blast Annotation	BLASTX Best Alignment Results				
					Total Score	Query Cover (%)	E-Value	Identity (%)	Accession
*GdauGR1*	Lack of 5′	168	55	gustatory receptor for sugar taste 64f-like [*Leptinotarsa decemlineata*]	67.4	35	8 × 10^−11^	50	XP_023027669.1
*GdauGR2*	Incomplete at both ends	675	224	gustatory receptor 11 [*Pyrrhalta aenescens*]	401	55	6 × 10^−135^	72.49	APC94336.1
*GdauGR3*	Lack of 5′	390	129	gustatory receptor 6 [*Pyrrhalta aenescens*]	268	84	9 × 10^−85^	72.43	APC94342.1
*GdauGR4*	Incomplete at both ends	735	244	gustatory receptor for sugar taste 64b-like [*Anoplophora glabripennis*]	164	91	4 × 10^−43^	37.84	XP_023312177.1
*GdauGR6*	Incomplete at both ends	240	79	gustatory receptor 14 [*Pyrrhalta aenescens*]	113	91	2 × 10^−27^	69.23	APC94341.1
*GdauGR7*	Lack of 3′	633	210	gustatory receptor 6 [*Pyrrhalta aenescens*]	287	86	3 × 10^−92^	64.35	APC94342.1
*GdauGR9*	Incomplete at both ends	156	51	gustatory receptor for bitter taste 66a-like isoform X1 [*Diabrotica virgifera virgifera*]	83.2	75	1 × 10^−17^	44.19	XP_050505927.1
*GdauGR10*	Incomplete at both ends	1242	413	gustatory receptor 7 [*Pyrrhalta aenescens*]	559	66	0.0	90.14	APC94345.1
*GdauGR11*	Incomplete at both ends	195	64	gustatory receptor 4 [*Pyrrhalta aenescens*]	133	98	1 × 10^−36^	84.51	APC94337.1
*GdauGR12*	Incomplete at both ends	252	83	gustatory receptor 1 [*Pyrrhalta maculicollis*]	111	59	3 × 10^−28^	63.86	APC94246.1
*GdauGR13*	Incomplete at both ends	252	83	putative gustatory receptor28b [*Diabrotica virgifera virgifera*]	106	47	1 × 10^−25^	51.11	XP_050507867.1
*GdauGR14*	Incomplete at both ends	288	95	gustatory receptor 1 [*Pyrrhalta aenescens*]	124	63	2 × 10^−33^	61.80	APC94331.1
*GdauGR15*	Incomplete at both ends	195	64	putative gustatory receptor 28b [*Diabrotica virgifera virgifera*]	123	71	2 × 10^−33^	57.61	XP_050507867.1
*GdauGR16*	Incomplete at both ends	315	104	gustatory receptor 10 isoform X2 [*Apis mellifera*]	77.0	66	4 × 10^−13^	44.21	XP_006567173.2
*GdauGR17*	Incomplete at both ends	618	205	gustatory and odorant receptor 22-like [*Anoplophora glabripennis*]	506	80	2 × 10^−175^	77.81	XP_018563765.1
*GdauGR18*	Incomplete at both ends	150	49	gustatory receptor for sugar taste 64a-like [*Diabrotica virgifera virgifera*]	108	100	2 × 10^−25^	54.84	XP_050518350.1
*GdauGR19*	Incomplete at both ends	117	38	gustatory receptor 8 [*Pyrrhalta aenescens*]	143	97	2 × 10^−40^	81.25	APC94346.1
*GdauGR20*	Incomplete at both ends	270	89	gustatory receptor 11 [*Pyrrhalta aenescens*]	399	63	3 × 10^−135^	72.12	APC94336.1
*GdauGR22*	Incomplete at both ends	180	59	putative gustatory receptor 28b [*Diabrotica virgifera virgifera*]	84	35	1 × 10^−15^	75.51	XP_028135243.2
*GdauGR23*	Incomplete at both ends	294	97	gustatory receptor 5a for trehalos [*Diabrotica virgifera virgifera*]	161	98	3 × 10^−45^	64.91	XP_050518348.1
*GdauGR24*	Incomplete at both ends	234	77	gustatory receptor 68a-like [*Diabrotica virgifera virgifera*]	130	88	3 × 10^−37^	80.77	XP_050504649.1
*GdauGR25*	Incomplete at both ends	213	70	gustatory receptor 3 [*Pyrrhalta maculicollis*]	148	63	5 × 10^−42^	77.78	APC94248.1
*GdauGR26*	Incomplete at both ends	234	77	gustatory receptor 1 [*Monochamus saltuarius*]	79.7	39	1 × 10^−15^	48.68	QUP79577.1
*GdauGR28*	Lack of 3′	1206	401	gustatory receptor 6 [*Pyrrhalta aenescens*]	546	97	0.0	67.58	APC94342.1
*GdauGR29*	Incomplete at both ends	258	85	gustatory receptor 1 [*Pyrrhalta aenescens*]	114	59	2 × 10^−29^	87.34	APC94331.1
*GdauGR30*	Incomplete at both ends	183	60	gustatory receptor 12 [*Pyrrhalta aenescens*]	118	59	8 × 10^−30^	90.32	APC94339.1

**Table 2:** Electrophysiological response frequency of gustatory sensilla in male and female adult *G. daurica*.

Treatment Group		Electrophysiological Response Frequency (Spikes/s)			
		Female Antennae	Male Antennae	Female Mouthpiece	Male Mouthpiece
PRU	0.1 mg/mL	5.00 ± 0.45 b	1.20 ± 0.37 b	1.60 ± 0.68 b	9.60 ± 1.44 b
	1.0 mg/mL	25.20 ± 2.94 a	24.60 ± 0.60 a	0.00 ± 0.00 b	17.60 ± 2.62 a
	10.0 mg/mL	0.00 ± 0.00 b	0.00 ± 0.00 b	30.40 ± 2.11 a	0.00 ± 0.00 c
SCU	0.1 mg/mL	1.00 ± 0.45 c	21.60 ± 1.54 b	1.20 ± 0.58 a	10.00 ± 1.30 a
	1.0 mg/mL	12.20 ± 0.97 b	28.60 ± 2.48 a	0.00 ± 0.00 b	0.00 ± 0.00 b
	10.0 mg/mL	19.20 ± 1.98 a	1.00 ± 0.45 c	0.00 ± 0.00 b	10.80 ± 0.97 a
NAR	0.1 mg/mL	1.40 ± 0.60 b	3.00 ± 0.45 b	1.00 ± 0.63 b	1.20 ± 0.37 a
	1.0 mg/mL	14.60 ± 2.66 a	15.2 ± 2.06 a	23.20 ± 1.62 a	0.00 ± 0.00 b
	10.0 mg/mL	19.00 ± 1.67 a	0.00 ± 0.00 b	0.00 ± 0.00 b	1.00 ± 0.32 a
RUT	0.1 mg/mL	1.00 ± 0.45 b	1.40 ± 0.40 b	0.00 ± 0.00 b	0.00 ± 0.00 b
	1.0 mg/mL	27.80 ± 1.07 a	5.00 ± 0.45 a	0.00 ± 0.00 b	1.40 ± 0.51 a
	10.0 mg/mL	0.00 ± 0.00 b	0.00 ± 0.00 c	25.40 ± 1.72 a	1.80 ± 0.58 a
ISO	0.1 mg/mL	14.80 ± 1.16 b	0.00 ± 0.00 c	0.00 ± 0.00 c	5.60 ± 0.68 c
	1.0 mg/mL	12.40 ± 0.68 b	6.20 ± 0.58 b	39.80 ± 1.39 b	43.20 ± 1.46 a
	10.0 mg/mL	66.20 ± 4.12 a	11.80 ± 0.66 a	48.40 ± 0.75 a	10.60 ± 1.08 b
IQC	0.1 mg/mL	7.00 ± 1.00 a	2.60 ± 0.51 b	0.00 ± 0.00 c	1.20 ± 0.58 a
	1.0 mg/mL	1.60 ± 0.68 b	51.80 ± 3.18 a	16.40 ± 1.72 a	1.00 ± 0.32 a
	10.0 mg/mL	9.00 ± 1.05 a	0.00 ± 0.00 b	8.40 ± 0.81 b	0.00 ± 0.00 a
PBG	1.0 mg/mL	1.00 ± 0.45 c	6.60 ± 0.93 b	0.00 ± 0.00 b	0.00 ± 0.00 a
	10.0 mg/mL	33.80 ± 1.20 b	0.00 ± 0.00 c	2.60 ± 0.93 b	0.00 ± 0.00 a
	100.0 mg/mL	38.00 ± 1.82 a	12.00 ± 0.95 a	32.60 ± 1.91 a	0.00 ± 0.00 a
TRE	10.0 mg/mL	4.80 ± 0.97 a	10.80 ± 0.97 a	0.00 ± 0.00 b	0.00 ± 0.00 a
	100.0 mg/mL	2.60 ± 0.60 b	1.00 ± 0.45 b	0.00 ± 0.00 b	0.00 ± 0.00 a
	1000.0 mg/mL	0.00 ± 0.00 c	0.00 ± 0.00 b	11.80 ± 0.86 a	0.00 ± 0.00 a
Gal	0.1 mg/mL	0.00 ± 0.00 a	3.60 ± 0.25 a	0.00 ± 0.00 a	0.00 ± 0.00 a
	1.0 mg/mL	0.00 ± 0.00 a	0.00 ± 0.00 b	0.00 ± 0.00 a	0.00 ± 0.00 a
	10.0 mg/mL	0.00 ± 0.00 a	0.00 ± 0.00 b	0.00 ± 0.00 a	0.00 ± 0.00 a
Rha	0.1 mg/mL	1.00 ± 0.32 a	0.00 ± 0.00 a	0.00 ± 0.00 a	0.00 ± 0.00 a
	1.0 mg/mL	0.00 ± 0.00 b	0.00 ± 0.00 a	0.00 ± 0.00 a	0.00 ± 0.00 a
	10.0 mg/mL	0.00 ± 0.00 b	0.00 ± 0.00 a	0.00 ± 0.00 a	0.00 ± 0.00 a

Notes: Data in the table are mean ± SD. Different lowercase letters after same column data indicate significant difference (*p* < 0.05), and same lowercase letters indicate no significant difference (*p* > 0.05).

## Data Availability

The original contributions presented in this study are included in the article/Supplementary Materials. Further inquiries can be directed to the corresponding author.

## Supplementary Materials

The following supporting information can be downloaded at: https://www.mdpi.com/article/10.3390/insects17040442/s1, Table S1: List of chemical and solvent information; Table S2: *Gdau*GRs primers of qRT-PCR; Table S3: Content determination of carbohydrate in *A. mongolicum*; Table S4: Content Determination of flavonoids in *A. mongolicum*; Table S5: Concentrations of ten test compounds used in single sensillum recording experiments; Table S6: Concentrations of six test compounds in the feeding assay.
