# Supplementary material for: Expression Characteristics of Gustatory Receptor Genes in Galeruca daurica (Coleoptera: Chrysomelidae) and Adult Behavioral and Electrophysiological Responses to Host Metabolites
Source: Insects. 2026 Apr 21;17(4):442. doi: 10.3390/insects17040442 (PMC13116256; doi:10.3390/insects17040442)
Supplement: Supplementary file 1 [file insects-17-00442-s001.zip › Table S1. List of Chemical and solvent information.pdf]

Table S1. List of Chemical and solvent information

Chenmical substance name	Molecular formula	Molecular weight	CAS number	Manufacturer	Purity
Prunin	$C_{21}H_{22}O_{10}$	434.39	529-55-5	Shanghai Aladdin Biochemical Technology Co., Ltd.	95%
Scutellarin	$C_{21}H_{18}O_{12}$	462.37	27740-01-8	Shanghai Shifeng Biotechnology Co., Ltd.	98%
Narcissoside	$C_{28}H_{32}O_{16}$	624.54	604-80-8	Shanghai Shifeng Biotechnology Co., Ltd.	98%
Rutin	$C_{27}H_{30}O_{16}$	610.52	153-18-4	Shanghai Yuanye Biotechnology Co., Ltd.	95%
Isoflavone	$C_{15}H_{10}O_{2}$	222.24	574-12-9	Beijing Boao Tuoda Technology Co., Ltd.	40%
Isoquercetin	$C_{21}H_{20}O_{12}$	464.38	482-35-9	Shanghai Yuanye Biotechnology Co., Ltd.	98%
Phenyl-β-D-glucopyranoside	$C_{12}H_{16}O_{6}$	256.25	1464-44-4	Shanghai Yuanye Biotechnology Co., Ltd.	98%
Trehalose	$C_{12}H_{22}O11$	342.30	99-20-7	Shanghai Aladdin Biochemical Technology Co., Ltd.	99%
D-Galactose	$C_{6}H_{12}O_{6}$	180.16	59-23-4	Shanghai Aladdin Biochemical Technology Co., Ltd.	99%
L-Rhamnose	$C_{6}H_{14}O_{6}$	182.17	10030-85-0	Shanghai Aladdin Biochemical Technology Co., Ltd.	99%
Anhydrous ethanol	$C_{2}H_{6}O$	46.07	64-17-5	Tianjin Sci-Tech Industrial Park Kemao Chemical Reagent Co., Ltd.	99.7%
