# Supplementary material for: Expression Characteristics of Gustatory Receptor Genes in Galeruca daurica (Coleoptera: Chrysomelidae) and Adult Behavioral and Electrophysiological Responses to Host Metabolites
Source: Insects. 2026 Apr 21;17(4):442. doi: 10.3390/insects17040442 (PMC13116256; doi:10.3390/insects17040442)
Supplement: Supplementary file 1 [file insects-17-00442-s001.zip › Table S2. GdauGRs primers of qRT-PCR.pdf]

Table S2. *Gdau*GRs primers of qRT-PCR.

Gene names	Forward primer（5′ to 3′）	Reverse primer（5′ to 3′）
SDHA	GGGAGACCACAATCTCCTCA	AGCTGGTGCTCCTAAGTCCA
GdauGR1	TCTCGTTTATTTCAACTGGCGA	ACCCAGAATTTCACCAACCAG
GdauGR2	ACACTTGGTCGAGCTTTCCT	GGAAACGACAGCTGCCAATT
GdauGR3	AAAGTTCGCTGCAATTATACCCA	GCAGCAATATTTAGTGTCAAACG
GdauGR4	CGCCCAATTGAAATTTCCGC	GGTTGTGTTGGTGTTCTCGG
GdauGR6	ACAGTGCAAGTTGTAATTGTGAA	CCACGAAGAAACCAGATGCC
GdauGR7	GGCTTTCCAATTCTATCCCGT	GGCATATACGGCGAGTGTTA
GdauGR9	TACCTGCATCTTTGGCCTCA	TCATGTCAGTTGAGCACGGA
GdauGR10	CTGCGGGGCTTTTATCACTT	TGGCCGTCATCCACAAGTAT
	GAGCTTGGTTGCACGATGAA	CCGATCCCGTGATTCCTATGG
GdauGR11	TGTGACCTGGAATTCGTTTGT	GGTAACCCGAGGAGATTTTCT
GdauGR12	TCTTATCTAGCGATGGGGTCC	CTCAAAGAATCCTGCTGCTGA
GdauGR13	CACAAAGTGGAAGCTAACGC	TGACTATAAAGAAGCCCGCTG
GdauGR14	CGTGCGACAAAGTGGAGAAA	TGACGTCAAAGAAGCCAGC
GdauGR15	ACTTGGGGAATTACGCTGAGA	TCACAATTGATTCCGTCTCGC
GdauGR16	GACGACGATCCGACAACAGA	CAGTTCTCTTTGTGCTTCGC
GdauGR17	TGGCTAGCGTTTATTGTCTGG	GGTGGATTGAGACGTATCGC
	TTGTGGGATGTGAAAGGGTTGA	CCAGAAATGCGGGACAAGAA
GdauGR18	TGGGAAATCTGGGAATACTGCT	CGAGCAAGAATCCAAAGGCA
GdauGR20	CATTATCATGCGACTTCACTGC	CCAAGTAAAGTGAAGTTCGGCA
	GTTGTGCGAAGAAGCTAACGA	AGCCCAACCAAATAGATCGCT
GdauGR22	TCACTGGTTTATCTATCTGCACA	TCTGGTGATTCTCGCTCAATT
GdauGR23	ACGATTATGTTTATTGCCGGAGT	GTATTTGACAGCTTCCGTAATGT
GdauGR24	GAGCCATCCGATTAGCAAAATT	TGAATACTGGAATCGTCTGTTGA
GdauGR25	GTGGAAAACGGAGGAAGACA	AGCGGAAAATATCGACGTCA
GdauGR26	ATGACTACAGAATCAGAACAGCT	AGTAAGTTGTGAGAGTGCCAA
GdauGR28	GCCGTATATGCCGTTGGTAC	CGCACACTCCCCAACTTATG
GdauGR29	TGTGATAGTCATCCTTTCTTGCG	TGCAGCTGAGAATTTTGGCC
GdauGR30	TTGAGAGCGACGGAAATGAA	GACTGCCATCTTCCGGAATG
