# Supplementary material for: Expression Characteristics of Gustatory Receptor Genes in Galeruca daurica (Coleoptera: Chrysomelidae) and Adult Behavioral and Electrophysiological Responses to Host Metabolites
Source: Insects. 2026 Apr 21;17(4):442. doi: 10.3390/insects17040442 (PMC13116256; doi:10.3390/insects17040442)
Supplement: Supplementary file 1 [file insects-17-00442-s001.zip › Table S3. Content Determination of Carbohydrate in A. mongolicum.pdf]

Table S3. Content Determination of Carbohydrate in *A. mongolicum*

Compounds	Class	CAS	Formula	Molecular Weight	Content of substances (mmol/g)
D-Ribono-1,4-lactone	monosaccharide	5336-08-3	$C_5H_8O_5$	148.0372	787871.0693
Deoxyglucose	monosaccharide	154-17-6	$C_6H_{12}O_5$	164.0685	639680.9152
D-Xylulose	monosaccharide	551-84-8	$C_5H_{10}O_5$	150.0528	488468.2400
Phenylglucoside	disaccharide	1464-44-4	$C_{12}H_{16}O_6$	256.0947	375498.3021
1,5-Anhydroglucitol	monosaccharide	154-58-5	$C_6H_{12}O_5$	164.0685	346071.0373
D-Ribose	monosaccharide	50-69-1	$C_5H_{10}O_5$	150.0528	298987.1156
D-Arabinitol	monosaccharide	488-82-4	$C_5H_{12}O_5$	152.0685	280501.4160
Xylitol	monosaccharide	87-99-0	$C_5H_{12}O_5$	152.0685	206843.6346
L-Rhamnose	monosaccharide	3615-41-6	$C_6H_{12}O_5$	164.0685	196760.6605
D-Arabinose	monosaccharide	10323-20-3	$C_5H_{10}O_5$	150.0528	183558.8996
Barium D-ribose-5-phosphate	monosaccharide	15673-79-7	$C_5H_9BaO_8P$	228.0035	154955.2506
D-Mannose-6-phosphate sodium salt	monosaccharide	70442-25-0	$C_6H_{12}NaO_9P$	273.0250	128361.7347
D-Glucuronic acid	monosaccharide	6556-12-3	$C_6H_{10}O_7$	194.0427	119886.2688
L-Fucose	monosaccharide	2438-80-4	$C_6H_{12}O_5$	164.0685	102133.9371
2-Acetamido-2-deoxy-D-glucopyranose	monosaccharide	7512-17-6	$C_8H_{15}NO_6$	221.0899	84790.1709
Maltose	disaccharide	69-79-4	$C_{12}H_{22}O_{11}$	342.1162	80443.5305
Levoglucosan	monosaccharide	498-07-7	$C_6H_{10}O_5$	162.0528	39661.0964
D-Mannose	monosaccharide	3458-28-4	$C_6H_{12}O_6$	180.0634	29611.5582
Cellobiose	disaccharide	528-50-7	$C_{12}H_{22}O_{11}$	342.1162	24496.1185
D-Xylose	monosaccharide	58-86-6	$C_5H_{10}O_5$	150.0528	22885.7462
Trehalose	disaccharide	99-20-7	$C_{12}H_{22}O_{11}$	342.1162	18291.4146
D-Galactose	monosaccharide	59-23-4	$C_6H_{12}O_6$	180.0634	11356.2539
Raffinose	trisaccharide	512-69-6	$C_{18}H_{32}O_{16}$	504.1690	9427.8861
Inositol	monosaccharide	87-89-8	$C_6H_{12}O_6$	180.0634	1855.9578
Glucose	monosaccharide	50-99-7	$C_6H_{12}O_6$	180.0634	106.7682
D-Fructose	monosaccharide	7660-25-5	$C_6H_{12}O_6$	180.0634	97.3267
Sucrose	disaccharide	57-50-1	$C_{12}H_{22}O_{11}$	342.1162	28.8553
