# Supplementary material for: Expression Characteristics of Gustatory Receptor Genes in Galeruca daurica (Coleoptera: Chrysomelidae) and Adult Behavioral and Electrophysiological Responses to Host Metabolites
Source: Insects. 2026 Apr 21;17(4):442. doi: 10.3390/insects17040442 (PMC13116256; doi:10.3390/insects17040442)
Supplement: Supplementary file 1 [file insects-17-00442-s001.zip › Table S4. Content Determination of Flavonoids in A. mongolicum.pdf]

Table S4. Content Determination of Flavonoids in *A. mongolicum*

Compounds	Class	CAS	Formula	Molecular Weight	Content of substances (after conversion)
Nicotiflorin	Flavones	17650-84-9	$C_{27}H_{30}O_{15}$	594.158475	0.0000612588
Rutin	Flavonols	153-18-4	$C_{27}H_{30}O_{16}$	610.15339	0.0000315038
Astragalin	Flavonols	480-10-4	$C_{21}H_{20}O_{11}$	448.100565	0.0000117059
Baimaside	Flavonols	18609-17-1	$C_{27}H_{30}O_{17}$	626.148305	0.0000108903
Apigenin-7-glucuronide	Flavones	29741-09-1	$C_{21}H_{18}O_{11}$	446.084915	0.0000041490
Miquelianin	Flavonols	22688-79-5	$C_{21}H_{18}O_{13}$	478.074745	0.0000040933
Kaempferol	Flavonols	520-18-3	$C_{15}H_{10}O_{6}$	286.04774	0.0000036010
Afzelechin	Flavonols	2545-00-8	$C_{15}H_{14}O_{5}$	274.084125	0.0000021153
Narcissin	Flavones	604-80-8	$C_{28}H_{32}O_{16}$	624.16904	0.0000012385
Quercetin	Flavonols	117-39-5	$C_{15}H_{10}O_{7}$	302.042655	0.0000011748
Phlorizin	Chalcones	60-81-1	$C_{21}H_{24}O_{10}$	436.13695	0.0000010484
Quercimeritrin	Flavonols	491-50-9	$C_{21}H_{20}O_{12}$	464.09548	0.0000008544
Scutellarin	Flavones	27740-01-8	$C_{21}H_{18}O_{12}$	462.07983	0.0000007848
Isorhamnetin 3-O-glucoside	Flavonols	5041-82-7	$C_{22}H_{22}O_{12}$	478.11113	0.0000006969
Prunin	Flavanones	529-55-5	$C_{21}H_{22}O_{10}$	434.1213	0.0000006255
Apigenin 7-glucoside	Flavones	578-74-5	$C_{21}H_{20}O_{10}$	432.10565	0.0000003421
Dihydrokaempferol	Flavanonols	480-20-6	$C_{15}H_{12}O_{6}$	288.06339	0.0000003023
Taxifolin	Flavanonols	480-18-2	$C_{15}H_{12}O_{7}$	304.058305	0.0000002289
Trilobatin	Chalcones	4192-90-9	$C_{21}H_{24}O_{10}$	436.13695	0.0000002204
Isorhamnetin	Flavonols	480-19-3	$C_{16}H_{12}O_{7}$	316.058305	0.0000001916
Spiraeoside	Flavonols	20229-56-5	$C_{21}H_{20}O_{12}$	464.09548	0.0000001853
Pinocembrin	Flavanones	480-39-7	$C_{15}H_{12}O_{4}$	256.07356	0.0000001753
Chrysin	Flavones	480-40-0	$C_{15}H_{10}O_{4}$	254.05791	0.0000001297
Apigenin	Flavones	520-36-5	$C_{15}H_{10}O_{5}$	270.052825	0.0000001202
Avicularin	Flavonols	572-30-5	$C_{20}H_{18}O_{11}$	434.084915	0.0000001014
Spinosin	Flavone glycosides	72063-39-9	$C_{28}H_{32}O_{15}$	608.174125	0.0000000949
Quercetin3-O- (6''-galloyl) -β-D-galactopyranoside	Flavonols	53171-28-1	$C_{28}H_{24}O_{16}$	616.10644	0.0000000397
Engeletin	Flavanones	572-31-6	$C_{21}H_{22}O_{10}$	434.1213	0.0000000382
Naringenin chalcone	Chalcones	25515-46-2	$C_{15}H_{12}O_{5}$	272.068475	0.0000000352
Homoplantaginin	Flavones	17680-84-1	$C_{22}H_{22}O_{11}$	462.116215	0.0000000347
Eriodictyol	Flavanonols	552-58-9	$C_{15}H_{12}O_{6}$	288.06339	0.0000000260
6-Methylflavone	Flavones	29976-75-8	$C_{16}H_{12}O_{2}$	236.08373	0.0000000203
Genkwanin	Flavones	437-64-9	$C_{16}H_{12}O_{5}$	284.068475	0.0000000200
7,4'-Di-O-methylapigenin	Flavones	5128-44-9	$C_{17}H_{14}O_{5}$	298.084125	0.0000000188
Mangiferin	Xanthones	4773-96-0	$C_{19}H_{18}O_{11}$	422.084915	0.0000000171
Sakuranetin	Flavones	2957-21-3	$C_{16}H_{14}O_{5}$	286.084125	0.0000000141

Pedalitin	Flavones	22384-63-0	$C_{16}H_{12}O_7$	316.058305	0.0000000123
Phloretin	Chalcones	60-82-2	$C_{15}H_{14}O_5$	274.084125	0.0000000104
Luteolin	Flavones	491-70-3	$C_{15}H_{10}O_6$	286.04774	0.0000000100
Diosmetin	Flavones	520-34-3	$C_{16}H_{12}O_6$	300.06339	0.0000000092
Isoginkgetin	Biflavonoids	548-19-6	$C_{32}H_{22}O_{10}$	566.1213	0.0000000080
Amentoflavone	Biflavonoids	1617-53-4	$C_{30}H_{18}O_{10}$	538.09	0.0000000070
Benzylideneacetophenone	Chalcones	94-41-7	$C_{15}H_{12}O$	208.088815	0.0000000062
Vitexin	Flavone glycosides	3681-93-4	$C_{21}H_{20}O_{10}$	432.10565	0.0000000055
Acacetin	Flavones	480-44-4	$C_{16}H_{12}O_5$	284.068475	0.0000000052
(-)-Epicatechin	Flavanones	490-46-0	$C_{15}H_{14}O_6$	290.07904	0.0000000046
Tectochrysin	Flavones	520-28-5	$C_{16}H_{12}O_4$	268.07356	0.0000000042
Kaempferide	Flavonols	491-54-3	$C_{16}H_{12}O_6$	300.06339	0.0000000040
Calycosin-7-O-β-D-glucoside	Isoflavanones	20633-67-4	$C_{22}H_{22}O_{10}$	446.1213	0.0000000039
3,7-Di-O-methylquercetin	Flavonols	2068-02-2	$C_{17}H_{14}O_7$	330.073955	0.0000000005
5-Hydroxyflavone	Flavones	491-78-1	$C_{15}H_{10}O_3$	238.062995	0.0000000003
