# Supplementary material for: Expression Characteristics of Gustatory Receptor Genes in Galeruca daurica (Coleoptera: Chrysomelidae) and Adult Behavioral and Electrophysiological Responses to Host Metabolites
Source: Insects. 2026 Apr 21;17(4):442. doi: 10.3390/insects17040442 (PMC13116256; doi:10.3390/insects17040442)
Supplement: Supplementary file 1 [file insects-17-00442-s001.zip › Table S6. Concentrations of six test compounds in the feeding assay.pdf]

Table S6. Concentrations of six test compounds in the feeding assay

Treatment Group	Concentration I	Concentration II
Control Group	Distilled water	
PRU	0.1 mg/ mL	1.0 mg/ mL
SCU	0.05 mg/ mL	0.5 mg/ mL
NAR	0.05 mg/ mL	0.5 mg/ mL
RUT	0.1 mg/ mL	1.0 mg/ mL
PBG	5.0 mg/ mL	50 mg/ mL
TRE	0.5 mg/ mL	5.0 mg/ mL
